# A CD4+CD161+ T-Cell Subset Present in Unexposed Humans, Not Tb Patients, Are Fast Acting Cells That Inhibit the Growth of Intracellular Mycobacteria Involving CD161 Pathway, Perforin, and IFN-γ/Autophagy

**DOI:** 10.3389/fimmu.2021.599641

**Published:** 2021-02-26

**Authors:** Rui Yang, Ying Peng, Jiang Pi, Yidian Liu, Enzhuo Yang, Xiaona Shen, Lan Yao, Ling Shen, Robert L. Modlin, Hongbo Shen, Wei Sha, Zheng W. Chen

**Affiliations:** ^1^Clinic and Research Center of Tuberculosis, Shanghai Key Lab of Tuberculosis, Shanghai Pulmonary Hospital, Institute for Advanced Study, Tongji University School of Medicine, Shanghai, China; ^2^Wuhan YZY Biopharma Co., Ltd, Biolake, Wuhan, China; ^3^Department of Microbiology and Immunology, Center for Primate Biomedical Research, University of Illinois College of Medicine, Chicago, IL, United States; ^4^Department of Microbiology, Immunology and Molecular Genetics, University of California, Los Angeles, Los Angeles, CA, United States

**Keywords:** CD4+CD161+ T cells, killing intracellular bacteria, IFN-γ, perforin, tuberculosis

## Abstract

It remains undefined whether a subset of CD4+ T cells can function as fast-acting cells to control *Mycobacterium tuberculosis* (Mtb) infection. Here we show that the primary CD4+CD161+ T-cell subset, not CD4+CD161-, in unexposed healthy humans fast acted as unconventional T cells capable of inhibiting intracellular Mtb and BCG growth upon exposure to infected autologous and allogeneic macrophages or lung epithelial A549 cells. Such inhibition coincided with the ability of primary CD4+CD161+ T cells to rapidly express/secrete anti-TB cytokines including IFN-γ, TNF-α, IL-17, and perforin upon exposure to Mtb. Mechanistically, blockades of CD161 pathway, perforin or IFN-γ by blocking mAbs abrogated the ability of CD4+CD161+ T cells to inhibit intracellular mycobacterial growth. Pre-treatment of infected macrophages with inhibitors of autophagy also blocked the CD4+CD161+ T cell-mediated growth inhibition of mycobacteria. Furthermore, adoptive transfer of human CD4+CD161+ T cells conferred protective immunity against mycobacterial infection in SCID mice. Surprisingly, CD4+CD161+ T cells in TB patients exhibited a loss or reduction of their capabilities to produce perforin/IFN-γ and to inhibit intracellular growth of mycobacteria in infected macrophages. These immune dysfunctions were consistent with PD1/Tim3 up-regulation on CD4+CD161+ T cells in active tuberculosis patients, and the blockade of PD1/Tim3 on this subset cells enhanced the inhibition of intracellular mycobacteria survival. Thus, these findings suggest that a fast-acting primary CD4+CD161+T-cell subset in unexposed humans employs the CD161 pathway, perforin, and IFN-γ/autophagy to inhibit the growth of intracellular mycobacteria, thereby distinguishing them from the slow adaptive responses of conventional CD4+ T cells. The presence of fast-acting CD4+CD161+ T-cell that inhibit mycobacterial growth in unexposed humans but not TB patients also implicates the role of these cells in protective immunity against initial Mtb infection.

## Introduction

Tuberculosis (TB), caused by *Mycobacterium tuberculosis* (Mtb), remains the top killer among infectious diseases largely due to epidemics of HIV/AIDS and drug resistance (1). The World Health Organization estimates that there are 10.4 million new cases and 1.7 million deaths annually, including 0.4 million deaths in people with HIV infection (2). Vaccines usually provide one of the most cost-effective interventions to prevent death and morbidity from infectious diseases. Nevertheless, the current TB vaccine, Bacille Calmette–Guérin (BCG), only protects young children from severe disseminated TB, but not effectively protects against pulmonary TB in adults or drug-resistant TB (3, 4). The development of a better TB vaccine or vaccination approach requires precisely elucidating protective immune mechanisms in humans.

Protective immune mechanisms against TB infection remain largely undefined (5). The current paradigm underscores the important role for CD4+ T cells in mounting adaptive anti-TB immunity (6, 7). HIV/AIDS depletes CD4+ T cells increasing TB susceptibility and severity (8), although it is not known about the relative importance of HIV immune suppression versus CD4+ T-cell decline. Concurrently, studies in mice indicate that CD4+ T cells are required for immunity against high-dose Mtb infection (9–11). CD4+ T cells can evolve into Th1 effector cells producing IFN-γ/TNF-α for macrophage activation and subsequent control of TB infection (12). CD4+ Th17 immunity is also implicated in animal models of vaccination and TB (13, 14). It is also noteworthy that IFN-γ-independent CD4+ T-cell immunity to TB has been reported (15, 16). On the other hand, Ag-activated CD4+ T effector cells generated from exposed or infected individuals can function as cytotoxic T cells producing cytotoxic granules or inhibiting of intracellular Mtb bacilli (17, 18). However, the phenotypes and functional mechanisms of CTL-like CD4+ T-cell immunity are unclear.

As opposed to slow-acting adaptive CD4+ T cells responses, a fast-acting CD4+ T-cell subset that controls very early Mtb infection was implicated in our mechanistic study in nonhuman primates (NHP) (19). In this NHP study, we demonstrated that the depletion of CD4+ T cells by anti-CD4 mAb treatment led to very early Mtb dissemination (bacteremia) after pulmonary infection (19). This “early-protecting” CD4+ T-cell subset appeared to be different from slow conventional CD4+ T cells, as losses of the slow/late adaptive anti-TB immunity and T helper functions were seen in the late phase of CD4 depletion at endpoint 2 months after Mtb infection (19).

To date, it remains unknown whether primary CD4+ T-cell population in unexposed humans comprise a fast-acting unconventional T-cell subset that inhibit intracellular Mtb growth or control initial Mtb infection (19, 20). It is noteworthy that three major types of unconventional T cells: MAIT, iNKT and γδ T cells share expression of CD161 (21, 22). We therefore tested the hypothesis that primary CD4+ T cells expressing CD161 may be consistent with the early protective CD4+ T-cell subset implicated in our NHP study (19). Here, we demonstrated that a primary CD4+CD161+ T-cell subset, not CD4+CD161- T cells, in unexposed healthy donors, fast acts as unconventional T cells capable of inhibiting intracellular Mtb growth and controlling mycobacterial infection. We also illustrated the mechanism whereby the CD4+CD161+ T-cell subset inhibits mycobacterial growth, and found a loss of this mechanism in active TB patients.

## Materials and Methods

### Ethics Statement

The protocols for use of human blood samples for experimental procedures were evaluated and approved by institutional review boards for human subjects’ research and institutional biosafety committees at Shanghai Pulmonary Hospital. All subjects were adults and anonymized, signed written informed consents.

### Study Subjects

Unexposed healthy controls (average age 35.7 years, 13 males, 7 females) recruited in this study had records of BCG vaccination at birth without any history and evidence of TB or LTBI as shown by clinical evaluations and T-SPOTS (QuantiFERON test). Active TB patients (average age 46.5 years, 12 males, 8 females) were confirmed by positive culture of *M. tuberculosis*, radiological findings, and clinical symptoms. In another pilot study, three active TB patients were recruited. Among all participants, there is no evidence for hepatitis B virus (HBV), hepatitis C virus (HCV), or human immunodeficiency virus (HIV) infection and other infectious diseases or cancers.

### Cell Culture and Reagents

THP-1 cells (Cell Bank, Chinese Academy of Sciences) were grown in RPMI 1640 (Gibco) supplemented and 10% heat-inactivated fetal bovine serum (FBS, Gibco). Prior to infection, THP-1 cells were treated with 50 ng/ml Phorbol 12-myristate 13-actate (PMA, Sigma-Aldrich) for 48 h to induce differentiation into macrophages, then washed three times with pre-warmed PBS and maintained in antibiotic-free media at 37°C for further use.

Human peripheral blood mononuclear cells (PBMCs) were isolated by density gradient centrifugation using Ficoll-Paque PLUS medium (GE) from buffy coats prepared from the peripheral blood of healthy donors (from Blood Center of Shanghai Changhai hospital) or from peripheral blood from active TB patients in Shanghai Pulmonary Hospital. Adherent-monocytes were enriched by adherence on plastic culture plates for 2 h. Non-adherent cells were removed *via* vigorous washing using pre-warmed PBS three times. Human monocytes-derived macrophages (hMDM) were differentiated from adherent-monocytes in media containing RPMI1640 (Gibco), supplemented with 10% heat-inactivated fetal bovine serum (FBS, Gibco) and 50 ng/ml human M-CSF (Novoprotein) for 7 days.

The following neutralization antibodies and their corresponding isotype controls were used in antibody blocking assays: anti-human TNF-α (Clone 28401, R&D) and its isotype control (Clone 11711, R&D); anti-human IFN-γ (Clone 25718,R&D),anti-human perforin (Clone1001103, R&D) and its isotype control (Clone 20102, R&D); anti-human IL-17 (AF-317, Polyclonal Goat IgG, R&D), anti-human granulysin (Polyclonal Goat IgG) and its isotype control (AB-108,R&D); anti-human FasL (Clone NOK-1,Biolegend) and its isotype control (Clone MOPC-21, Biolegend); anti-human PD1 (Clone EH12.2H7, Biolegend), and anti-human Tim3 (Clone F38-2E2, Biolegend). The blocking antibodies to CD161 were purchased from BD (Clone DX12) and Miltenyi (Clone 191B8) respectively. Autophagy inhibitor, 3-MA, was purchased from Beyotime.

### Purification of CD4+CD161+ cells, CD4+CD161-Cells and CD20+B Cells

**Figure 1:**
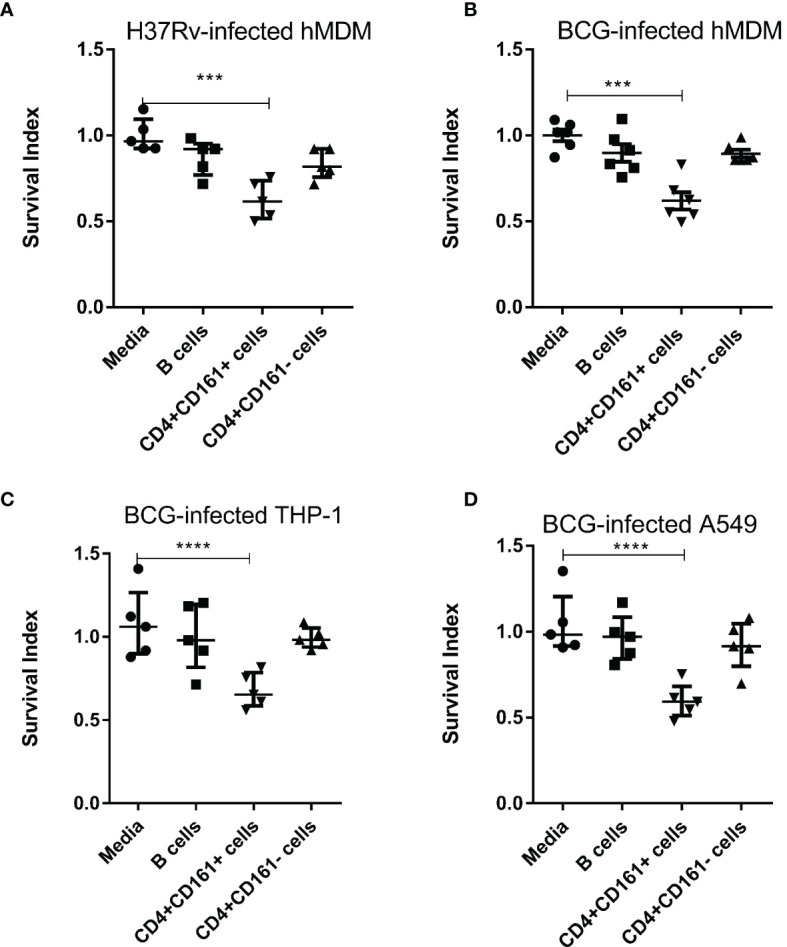
PrimaryCD4+CD161+ T-cell subset, notCD4+CD161-, from unexposed healthy donors rapidly mediates inhibition of Mtb H37Rv and M. bovis BCG in autologous or allogeneic macrophages and lung epithelial cells. **(A)** Shows that primary CD4+CD161+ T cells, notCD4+CD161- or B cells controls, significantly restricted intracellular growth of Mtb H37Rvin infected autologous hMDM. Shown are Survival Index derived from the calculation: = 100 x CFU of co-cultured group/CFU of infected group, as described in Methods. **(B–D)** show that primaryCD4+CD161+ T cells, not controls, similarly inhibited intracellular *M. bovis* BCG growth in infected autologous hMDM **(B)**, THP-1 macrophages **(C)** and A549 lung epithelial cells **(D)**. CD4+CD161+ T cells,CD4+CD161- T cells, and CD20+ B cells were purified from PBMC of unexposed healthy donors as indicated in the Methods section and **Suppplementary Figure 1A**, co-cultured with Mtb H37Rv-infected hMDM **(A)** or BCG-infected hMDM **(B)**, THP-1 **(C)** or A549 **(D)** at a ratio of 10:1 for 72 h. Viable mycobacteria in each cell lysate were quantified by counting colony-forming unit (CFU). CFU counts of Mtb H37Rv or BCG bacilli for each group were normalized by those of infected/media group in each experiment. Data shown as median ± IQR are pooled from five **(A, C, D)**, six **(B)** independent experiments. Each experiment involved 2-5 healthy donors. ****p < 0.0001, ***p < 0.001 vs. Media group (ANOVA, Dunnett’s test).

CD4+ T cells were enriched from PBMC of healthy donors or active TB patients by negative selection method using CD4+ T Cell Isolation Kit II from Miltenyi (130-096-533). Then, CD4+ T cells were stained with anti-human CD161-APC (BD, 550968), followed by anti-APC magnetic beads (Miltenyi Biotech, 130-090-855) for secondary positive purification. Cells were loaded onto columns and the passing cells were collected as CD4+CD161- T cells, CD4+CD161+ T cells were released from columns using release buffer (Miltenyi Biotech). There was no evidence for significant activation ofCD4+CD161+ T cells after purification (data not shown). B cells were positivity isolated from PBMC using anti-CD20 microbeads from Miltenyi Biotech according to the manufacturer’s instructions. Cell purity was consistently ≥96% as demonstrated in **Suppplementary Figure 1A**.

### Mycobacteria Culture, Infection of Cells and Quantification of Intracellular Mycobacterial Growth

*Mycobacterium bovis*, Bacillus Calmette-Guerin (BCG) and *M. tuberculosis* H37Rv were grown into log phase at 37°C in Difco Middlebrook 7H9 broth medium (Becton Dickinson) with 10% oleic acid-albumin-dextrose-catalase (OADC) Enrichment (Becton Dickinson), 0.05% (v/v) Tween 80 and 0.2% (v/v) glycerol. Basically, cells were infected with BCG at a multiplicity-of-infection (MOI) of 10 bacilli to one cell overnight served as target cells. Human monocyte-derived macrophage (hMDM) cells were infected with H37Rv at a MOI of four for 4 h. After infection, extracellular non-internalized bacilli were removed by washing with pre-warmed PBS four times. Then, 1 X 10^3^ mycobacteria-infected THP-1, A549, or hMDM cells were co-cultured with purified CD4+CD161+/CD4+CD161- T and B cells at a ratio of 1:10 in the presence or absence of neutralization Abs (5 ug/ml) and their isotype controls, or inhibitors (inhibitors were pre-incubated with cells for 2 h) in 200 ul media without antibiotic in 96-well-plates for 3 days. Then, wells were aspirated, and the infected cells were lysed in 200 ul of sterile PBS with 0.067% SDS. A 10-fold serial dilution was performed for quantitative measurement of viability. 100ul of aliquots were platted in triplicate on Middlebrook 7H10 or 7H11 agar plates supplied with 10% OADC for 2–3 weeks until colonies were large enough to be counted. The percentage Survival index (23) represented as intracellular bacteria survival was calculated as follows: Survival index = 100 x CFU of co-cultured group/CFU of infected group.

### Adoptive Transfer of CD4+CD161+ and CD4+CD161- T Cells to BCG-Infected SCID Mice

Four-week-old female SCID mice (Shanghai SLAC Laboratory Animal) were infected intravenously *via* the tail vein with 1 x 10^7^ CFU of BCG in 0.2 ml PBS. After 3 days, 5 × 10^5^ sorted human CD4+CD161+ T cells, CD4+CD161- T cells or PBS were transferred into BCG-infected recipient mice (n=8 for each group) in 0.2 ml PBS through intravenously *via* the tail vein. At day 25 post-infection, the lungs of all mice were harvested, homogenized in PBS, and plated on 7H10 agar at 10-fold series dilution to enumerate BCG bacilli.

### Flow Cytometry Analysis

For cell frequency analysis, cells were incubated with PB-anti-CD3 (SP34-2, BD), FITC-anti-CD4 (SK3, BD), BV605-anti-CD161 (HP3G8, Biolegend). For analysis of the cell memory state, cells were incubated with PB-anti-CD3 (SP34-2, BD), FITC-anti-CD4 (SK3, BD), BV605-anti-CD161 (HP3G8, Biolegend), BV711-anti-CCR7 (G043H7, Biolegend), PE/Cy7-anti-CD45RA (HI100, Biolegend) for 20 min at room temperature in dark. For phenotyping of special surface markers, cells were incubated with PB-anti-CD3 (SP34-2, BD), FITC-anti-CD4 (7A5, Thermo Scientific), APC-anti-CD161 (B6, Biolegend), AF700-anti-Tim-3 (J418F1, Biolegend), BV605-anti-PD-1 (TS1/18, Biolegend) for 20 min at room temperature in dark. For functional assay, PBMC were stimulated with PMA (50 ng/ml) + ionomycin (1 μg/ml) (1 h) and brefeldin A (10 μg/ml) were added for 5 h. Cells were stained with monoclonal antibodies to the surface markers, PB-anti-CD3 (SP34-2, BD), FITC-anti-CD4 (SK3, BD), BV605-anti-CD161 (HP3G8, Biolegend), for 30 min at room temperature in the dark. After washing twice, cells were fixed in fixation/permeabilization buffer (BD), followed by intracellular cytokine staining with antibodies, BV711-anti-IFN-γ (4S.B3; Biolegend), PE/Cy7-anti-TNF-α (Mab11, Biolegend). For detection of perforin, PBMC were stimulated with BCG-infected hMDM cells for 8 h and brefeldin A (10 μg/ml) added for the last 4 h. Cells were stained with monoclonal antibodies to the surface markers, PB-anti-CD3 (SP34-2, BD), BV510-anti-CD4 (SK3, BD), BV605-anti-CD161 (HP3G8, Biolegend), for 30 min on ice in the dark. After washing twice, cells were fixed using fixation/permeabilization buffer (BD), followed by intracellular cytokine staining with FITC-anti-perforin (dG9; Biolegend). Then cells were acquired on an LSR Fortessa flow cytometer (BD), and the data were analyzed with FlowJo software (TreeStar).

### Quantification of Gene Expression by RT-qPCR

Sorted CD4+CD161+ orCD4+CD161- T cells from healthy controls were co-cultured with BCG-infected hMDM for 24 h. RNA isolation from isolated cells, reverse-transcription and PCR reactions were done as described in (24). Primers for IFNG, PRF and EF1A were reported in (25). Other primers used for amplification in this study were synthesized from Sangon Biotech: IL17A-F,5′-CGGACTGTGATGGTCAACCTGA -3′, IL17A-R, 5′-GCACTTTGCCTCCCAG ATCACA-3′; FASLG-F,5′-GGTTCTGGTTGCCTTGGTAGGA-3,FASLG-R, 5′-CTGTGTG CATCTGGCTGGTAGA -3′. EF1A was used as a reference control. Fold change was calculated with the ΔΔCT method.

### Measuring Secretory Cytokines Using ELISA

The amounts of IFN-γ and IL-17A in cell supernatants were detected using human cytokine ELISA kit (C608 for Human IFN-γ; C623 for Human IL-17A; Genstar) according to the manufacturer’s instructions.

### LC3B Staining

Cells were then fixed in 4% paraformaldehyde for 10 min, permeabilized with 0.2% Triton X-100/PBS for 10 min, and pre-blocked in 5% BSA/PBS overnight. The cells were then incubated with rabbit LC3B antibody that was diluted at 1/100 in blocking solution for 2 h, washed three times with PBS, and incubated with Alexa fluor 488-conjugated anti-rabbit IgG Ab (1/200 in blocking solution) for 1 h. After washing with PBS, the cells were further incubated with 10 μg/ml Hocheststain for 20 min, and then used for confocal microscopy (Zeiss, German) analysis.

### Statistical Analysis

Statistical analysis was performed with GraphPad Prism 6.0. Differences between groups were assessed by t test, nonparametric t test, or one-way ANOVA, followed by the Dunnett’s test or Tukey’s multiple comparison test indicated in each figure.

## Results

### A Primary CD4+CD161+ T-Cell Subset, Not CD4+CD161- T Cells, From Unexposed Healthy Humans Inhibited Intracellular Mycobacterial Growth in Infected Autologous and Allogeneic Macrophages as Well as Lung Epithelial Cells

Our recent CD4 depletion studies in NHPs suggest that CD4+ T cells contain a fast-acting T-cell subpopulation which is required to control very early Mtb dissemination after pulmonary infection (19). Notably, human CD161 is considered to be a surrogate marker of unconventional T cells, as it is expressed on three major types of such T cells: MAIT, iNKT and gamma delta T cells (21, 22). In addition, CD161 expression on an Foxp3^+^ Treg subset allows for the innate production of IL-17 and other pro-inflammatory cytokines (26, 27). Since NHPs closely resemble humans, we hypothesized that human CD4+CD161+ T cells may represent the fast-acting CD4+ T-cell subset required to control early Mtb dissemination after pulmonary infection in NHPs (19). To test this hypothesis, we assessed CD4+CD161+ T cells for the ability to inhibit intracellular Mtb growth using an *in vitro* infection model as described by us and others (23, 28). Thus, CD4+CD161+ T cells and controls, CD4+CD161- T and CD20+ B cells, were purified from PBMC of unexposed healthy humans using immune-magnetic bead-based standard methods. The isolated cell subsets were shown to be ≥96% pure (**Suppplementary Figure 1A**). Then, CD4+CD161+ T cells, as well as control CD4+CD161- T cells and B cells, were co-cultured, respectively, with Mtb-infected autologous monocyte-derived macrophages (hMDM) at a ratio of 10:1 for 3 days. Strikingly, CD4+CD161+ T cells, but not the CD4+CD161- T cell control, significantly inhibited intracellular Mtb growth in infected autologous hMDM (**Figure 1A**). We then employed *M. bovis* BCG-infected target cells for subsequent mechanistic experiments, as studies published by us and peers showed that T effector cells capable of restricting intracellular BCG replication can also similarly inhibit intracellular Mtb growth (29–31). While CD4+CD161+T cells, not controls, similarly mediated growth inhibition of BCG and Mtb in infected autologous hMDM (**Figures 1A, B**), they also inhibited intracellular BCG growth in the THP-1 human macrophages (**Figure 1C**). Concurrently, CD4+CD161+ T cells also inhibited intracellular BCG growth in BCG-infected A549 lung cells as well (**Figure 1D**). Since CD4+CD161+ T cells were derived from healthy donors express potential MHC II different from THP-1 or A549 target cells, such mismatches implicate that CD4+CD161+ T-cell inhibition of intracellular mycobacteria does not likely require MHC II-restricted antigen recognition. Taken together, these results suggest that primary CD4+CD161+ T-cell subset differ from CD4+CD161- T cells in the ability to directly inhibit mycobacterial growth in both autologous and allogeneic target cells.

### CD4+CD161+ T Cell-Mediated Inhibition of Mycobacterial Growth Coincided With the Rapid Expression of Anti-TB Cytokines Including IFN-γ, TNF-α, IL-17A and Perforin

**Figure 2:**
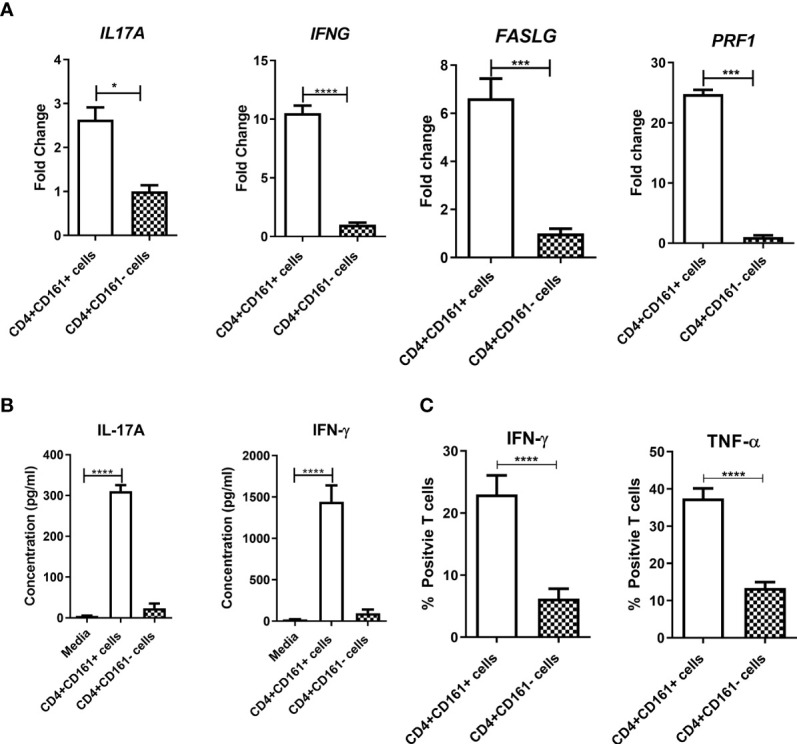
CD4+CD161+ T cell-mediated inhibition of mycobacterial growth coincided with the ability to rapidly express anti-TB cytokines including IFN-γ, TNF-α, IL-17A and perforin. Bar graph **(A)** shows the fold changes in expression levels of individual anti-TB cytokine genes in CD4+CD161+ T cells andCD4+CD161- controls co-cultured for 24 h with autologous BCG-infected hMDM determined by RT-qPCR. Data shown as mean ± SD are derived from five experiments. ****p < 0.0001, ***p < 0.001, *p < 0.05 (t test). Bar graph **(B)** shows the means± SD values of secreted IFN-γ and IL17A in the supernatant from the BCG-infected hMDM culture, theCD4+CD161+ T cell plus BCG-infected hMDM co-cultures and theCD4+CD161- plus infected hMDM co-culture. Data are pooled from 15 healthy donors. ****p < 0.0001 vs. Media group (ANOVA, Dunnett’s test). Bar graph **(C)** shows flow cytometry frequency data from ICS assay indicating thatCD4+CD161+ T cells exhibited stronger capability of producing Th1 cytokines IFN-γ and TNF-α. PBMC isolated from unexposed healthy donors (n=15) were stimulated with PMA/Ionomycin, then stained intracellularly for IFN-γ and TNF-α. ****p < 0.0001 (t test). The representative flow cytometry histograms with gating plots were presented in **Supplementary Figure 1B**.

The expression of CD161 on CD4+ T cells is implicated as a marker of Th17 cells producing IL-17 upon stimulation with cytokines and TCR engagement (32), but also is linked to IFN-γ production (33). However, it is not known whether CD4+CD161+ T cells could secrete anti-TB cytokines upon encountering infected target cells. Whether such secretory cytokines lead to mycobacterial growth inhibition is also not known. To address these questions, primary CD4+CD161+T cells or CD4+CD161-controls were co-cultured with BCG-infected hMDM for 24 h, and then assessed supernatants for expression/production of anti-TB cytokines. Indeed, CD4+CD161+T cells expressed significantly higher levels of IFN-γ and IL-17 transcripts than CD4+CD161- cells (**Figure 2A**). Concurrently, CD4+CD161+T cells also expressed much higher levels of antimicrobial CTL-related molecules perforin and Fas ligand than did CD4+CD161-controls (**Figure 2A**). We found that cultured CD4+CD161+T cells, but not CD4+CD161-controls, secreted large amounts of IFN-γ and IL-17 cytokines in the day 3 supernatants (**Figure 2B**). Particularly, the level of secreted IFN-γ was almost 1,500 pg/ml high, 5 times higher than IL-17, suggesting that CD4+CD161+T cells were Th1 dominant upon the exposure. To define this Th1 phenotype, we measured IFN-γ- and TNF-α-producing CD4+CD161+T cells using intracellular cytokine staining after stimulation with PMA and ionomycin. Intracellular cytokine staining demonstrated that >23% of CD4+CD161+T cells showed the Th1 phenotype producing anti-TB cytokines IFN-γ and IFN-α, whereas CD4+CD161- T cells exhibited a much lower Th1 frequency (**Figure 2C**, **Suppplementary Figure 1B**).

### Perforin and IFN-γ Produced by CD4+CD161+ T Cells Were Required to Inhibit Intracellular Mycobacterial Growth, Involving Autophagy in Infected hMDM

**Figure 3:**
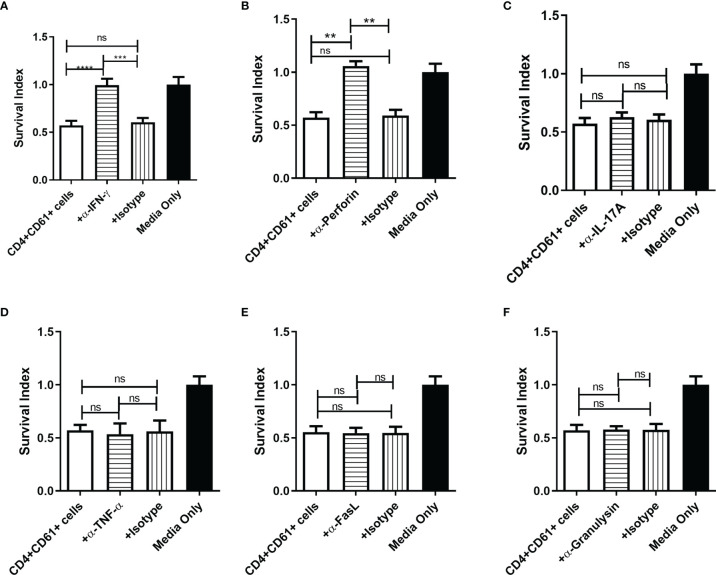
Perforin and IFN-γ produced byCD4+CD161+ T cells were required to inhibit intracellular mycobacterial growth. **(A–F)** Two panels of bar graphs show the effect of anti-cytokine neutralization Abs on CD4+CD161+ T-cell inhibition of intracellular BCG growth. Shown are mean survival indexes ± SD for BCG bacilli in infected hMDM (Media only) andCD4+CD161+ T cells (+ DP T cells) plus infected hMDM co-cultured for 72 h at a ratio of 10:1 in the absence or presence of anti-IFN-γ, anti-IL-17A, anti-TNF-α, anti-perforin, anti-granulysin, anti-FasL Abs, and corresponding Ig isotype controls, respectively (5 ug/ml for each). Note that only the anti-IFN-γ and anti-perforin blockades in the co-culture system could reverse theCD4+CD161+ T-cell-mediated inhibition of mycobacterial growth. Data shown as mean ± SD are derived from five experiments using 12 healthy donors for preparing hMDM. ****p < 0.0001, ***p < 0.001, **p < 0.05, ns, not significant (ANOVA, Tukey’s test).

We then sought to determine which of the cytokines produced by CD4+CD161+T cells contributed to the inhibition of intracellular mycobacterial growth in infected target cells. To address this, we performed blocking experiments using neutralizing anti-cytokine antibodies in the intracellular mycobacterial inhibition assay. To our surprise, blockade of IFN-γ and perforin could significantly reverse or abrogate the ability of primary CD4+CD161+ T cells to inhibit intracellular mycobacterial growth in infected hMDM (**Figures 3A, B**). However, neutralization of IL-17, TNF-α or Fas-L had little or no effects on the observed inhibition (**Figures 3C–F**). Adding anti-granulysin blocking Ab to the co-culture did not alter the inhibition, as CD4+CD161+T cells produced no or low-level granulysin (**Figure 3E** and data not shown).

**Figure 4:**
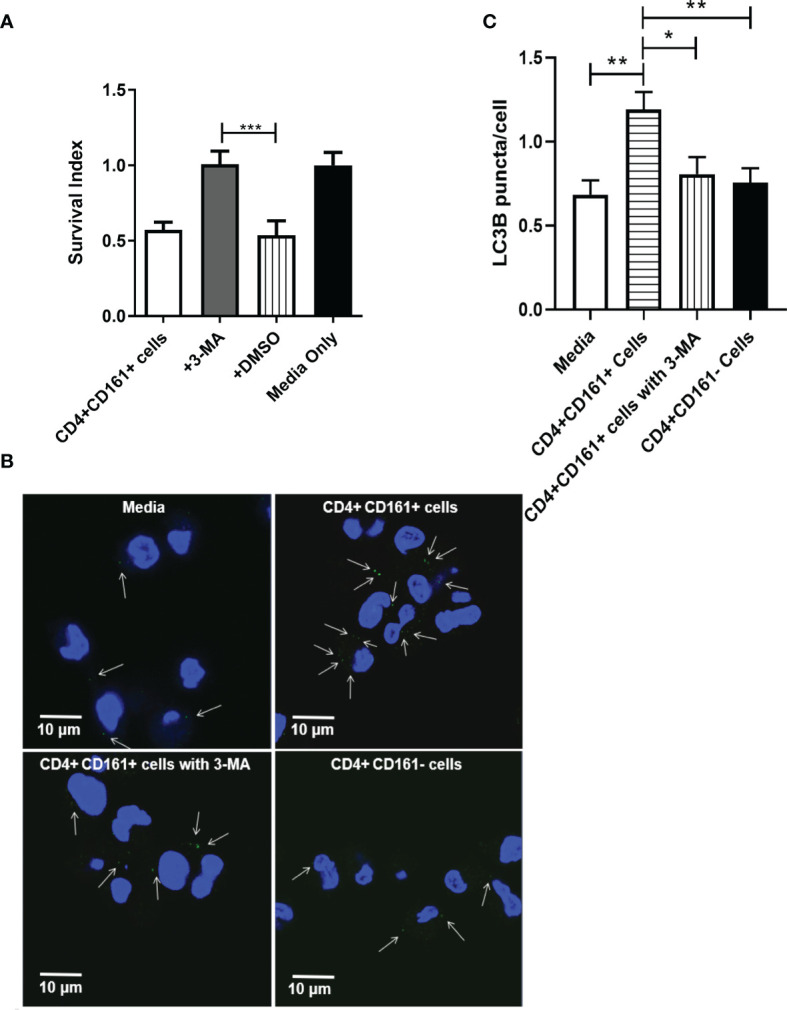
The inhibition of intracellular mycobacterial growth mediated by CD4+CD161+ T cells involved with autophagy in infected hMDM. Bar graph **(A)** shows that autophagy was involved inCD4+CD161+ T cell-mediated inhibition of intracellular mycobacterial growth. BCG-infected hMDM were pre-treated for 2 h with DMSO or 3-MA (10 uM), respectively, and then washed. These BCG-infected hMDMs were individually co-cultured for 72 h with CD4+CD161+ T cells or CD4+CD161- controls at a ratio of 10:1. Data shown as mean ± SD are derived from five experiments involving 10 healthy donors. ***p < 0.001 (ANOVA, Tukey’s test). Graph **(B)** shows thatCD4+CD161+ T cell could increase the LC3B puncta in BCG-infected hMDM. BCG-infected hMDM were pre-treated for 2 h with 3-MA (10 uM), and then washed. Then, these BCG-infected hMDMs were co-cultured for 72 h withCD4+CD161+ T cells orCD4+CD161- T cells at a ratio of 10:1. White arrows indicate the LC3B puncta. Bar graph **(C)** shows the statistical analysis of LC3B puncta per cell in BCG-infected hMDM. LC3B puncta in more than 150 cells were counted in each group. **Suppplementary Figure 1D** shown the specificity of LC3B staining in hMDM. *p < 0.05, **p < 0.01, (ANOVA, Tukey’s test).

Recent studies showed that IFN-γ could activate selective autophagy leading to inhibition of mycobacteria in infected hMDM (34). We therefore examined whether the selective autophagy process played a role in IFN-γ-producing CD4+CD161+ T-cell inhibition of intracellular mycobacteria in hMDM. In order to block autophagy, we preincubated hMDM prior to coculture with CD4+CD161+ T cells with 3-MA, which has been shown to inhibit autophagy sequestration (35). Notably, the 3-MA pre-treatment of BCG-infected hMDM significantly blocked the CD4+CD161+ T-cell restriction of intracellular mycobacterial growth, as compared to the DMSO control (**Figure 4A**). In parallel, we also found less LC3B puncta in the co-cultures with the 3-MA pre-treated hMDM (**Figures 4B, C**). Consistently, the addition of CD4+CD161+ T cells resulted in more LC3B puncta in hMDM as compared to the addition of CD4+CD161- T cells (**Figures 4B, C**). These results suggest that perforin and IFN-γ produced by CD4+CD161+T cells act in concert to inhibit intracellular mycobacterial growth, involving down-stream selective autophagy in infected macrophages.

### CD161 Blockade Reduced the Ability of CD4+CD161+T Cells to Inhibit Intracellular Mycobacterial Growth

**Figure 5:**
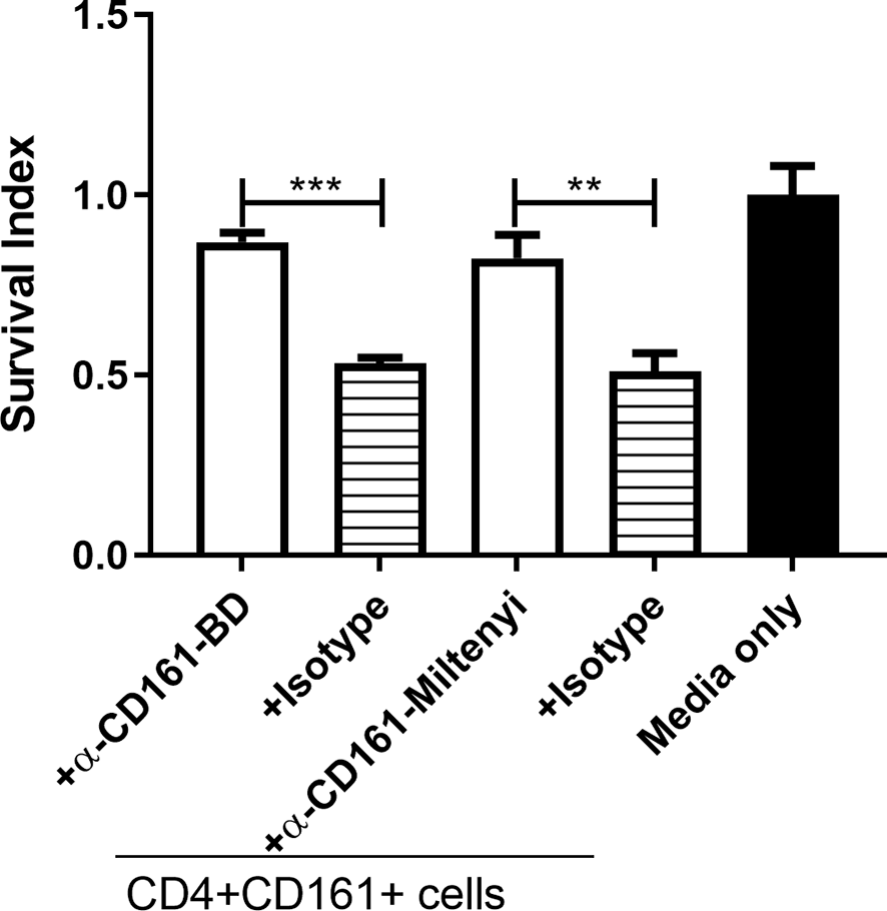
CD161 blockade reduced the ability of primaryCD4+CD161+T cells to inhibit intracellular mycobacterial growth. Shown are bar graph data of Survival indexes for BCG bacilli in infected hMDM alone or co-cultured with primaryCD4+CD161+ T cells plus infected hMDM co-cultured for 72 h at a ratio of 10:1 in the absence or presence of two anti-CD161 mAbs (5 ug/ml for each) or corresponding Ig isotype controls (5 ug/ml for each). Note that anti-CD161 neutralizing mAbs from two sources (BD and MACS), not isotype controls, could each reverse the ability of CD4+CD161+ T cells to inhibit intracellular mycobacterial growth. Data were pooled from three independent experiments involving 10 healthy donors. ***p < 0.001, **p < 0.01 (ANOVA, Tukey’s test).

The CD161 ligand, Letin-like transcript 1 (LLT1), is expressed on monocytes/macrophages and DC, and LLT1/CD161 signaling has been shown to stimulate Th1 differentiation (36). We hypothesized that the CD161 signal pathway contributes to the observed fast-acting antimicrobial function of CD4+CD161+T cells. To test this hypothesis, we performed CD161 blocking experiments using anti-CD161 neutralizing antibodies from two commercial sources. The neutralization activities of these two anti-CD161 antibodies have been documented in early reports (36). Interestingly, we found that antibody blockade of the CD161 pathway indeed reversed the CD4+CD161+T-cell inhibition of BCG growth in infected hMDM (**Figure 5**). These results suggest that CD161 signaling pathway play a role in the mycobacterial growth inhibition in infected macrophages.

### Adoptive Transfer of Human CD4+CD161+ T Cells Conferred Protective Immunity Against Mycobacterial BCG Infection in SCID Mice

**Figure 6:**
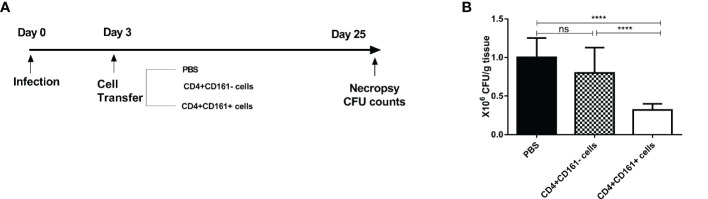
Adoptive transfer of primary humanCD4+CD161+ T cells conferred protective immunity against mycobacterial infection in SCID mice. Schematic diagram **(A)** shows the experimental strategy of adoptive transfer of CD4+CD161+ T cells or CD4+CD161- and PBS controls to BCG-infected SCID mice. Bar graph **(B)** compares bacterial burden in the lungs of the test and control groups of SCID mice (n = 8 per group). Note that the test group of animals receiving primary CD4+CD161+ T cells showed significantly lower CFU counts of BCG bacilli in the lungs than the control groups of mice receivingCD4+CD161- controls or PBS. ****p < 0.0001, ns, not significant (ANOVA, Tukey’s test).

Based on the *in vitro* findings as defined above, we hypothesized that CD4+CD161+T cells can function as fast-acting T cells to control mycobacterial infection *in vivo*. To test this hypothesis, we developed a cell adoptive transfer strategy using BCG-infected SCID mice as a model (**Figure 6A**). CD4+CD161+ and CD4+CD161- T cells were purified from PBMCs of unexposed healthy donors, and then adoptively transferred to SCID mice at day 3 after BCG infection (**Figure 6A**). This proof-of-concept study was focused on the immune control of bacillary burden after infection, as our recent NHP work demonstrated that this CD4+ T-cell subset is required to control very early Mtb dissemination (19). Interestingly, mice infused with CD4+CD161+ T cells showed significantly lower bacterial burdens in lungs, compared with control mice receiving CD4+CD161- T cells or PBS (**Figure 6B**). Thus, CD4+CD161+T cells, but not the CD4+CD161- T cell control, can confer immune control of mycobacterial infection in SCID mice. These findings suggest that CD4+CD161+T cells play a role in protective immunity against mycobacterial infection *in vivo*.

### Circulating CD4+CD161+ T Cells From TB Patients Failed to Inhibit Intracellular Mycobacterial Growth, Which Was Linked to a Reduced Ability to Produce IFN-γ and Perforin

**Figure 7:**
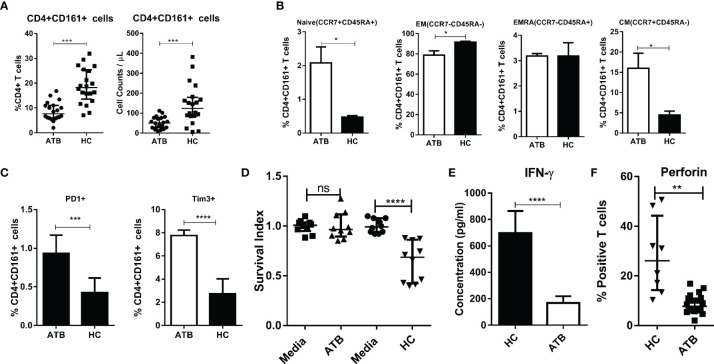
Circulating CD4+CD161+ T cells in TB patients failed to inhibit intracellular mycobacterial growth, exhibiting a reduced ability to produce IFN-γ and perforin. **(A)** Graphs compares the frequency and absolute counts of CD4+CD161+ T cells among total CD4 T cells between patients with active tuberculosis (ATB) and heathy donors. Representative flow cytometric histogram dot-plots are shown in **Supplementary Figure 1C**. Note that ATB patients (n=20) showed a significantly lower frequency and counts of CD4+CD161+ T cells in the blood than that of healthy controls (n=20). ***p < 0.001, (nonparametric t test). **(B)** Bar graph shows that mostCD4+CD161+ T cells in both ATB patients and healthy controls predominantly displayed the CCR7-CD45- effector memory (EM) T cell phenotype, with ~80 and ~95% of them being expressed in the ATB and control group, respectively, whereas CCR7+CD45RA- central memory (CM) T cells were higher ~15%) in the ATB group than that in controls (~10%). Representative flow cytometry histograms measuring the phenotypes are shown in **Supplementary Figure 1C**. **p < 0.01, *p < 0.05 (nonparametric t test). Bar graph **(C)** shows that ATB patients (n=10) had significantly higher expression levels of Tim-3 and PD-1 inCD4+CD161+ T cells than healthy donors (n=10). Representative flow cytometry histograms measuring the phenotypes are shown in **Supplementary Figure 1C**. *p < 0.05 (nonparametric t test). Bar graph **(D)** compares the mean Survival indexes ± SD of BCG in the co-cultures of infected hMDM and CD4+CD161+ T cells from ATB patients (n=10) or healthy donors (n=10). Note that CD4+CD161+ T cells purified from PBMC of ATB patients failed to control intracellular mycobacterial growth. **p < 0.01, *p < 0.05 (nonparametric t test). Bar graph **(E)** compares amounts of secretory IFN-γin the co-cultured conditions indicated above. Note that the amount of IFN-γ secreted by CD4+CD161+ T cells from ATB patients was significantly lower than that from healthy donors when simulated 24 h with BCG-infected autologous hMDM. ****p < 0.0001(nonparametric t test). Bar graph **(F)** shows that CD4+CD161+ T cells from ATB patients (n=8) expressed lower Perforin than those from healthy controls (n=8) when stimulated with BCG-infected autologous hMDM. Representative flow cytometry histogram measuring perforin-producing cells are shown in **Supplementary Figure 1D**. **p < 0.01 (t test).

Finally, we sought to examine whether there were alternations in the phenotype and function of CD4+CD161+T cells in ATB patients. Consistent with the decreased CD161 expression in CD4+ T cells in ATB cohorts (37), the frequency of CD4+CD161+ T cells in the blood of ATB patients was significantly lower than those in healthy controls (**Figure 7A**, **Suppplementary Figure 1C**). Notably, of the primary CD4+CD161+T cells in both ATB patients and healthy controls, the majority, ~80 and ~95%, respectively, displayed the CCR7-CD45- effector memory (EM) T cell phenotype (**Figure 7B**, **Suppplementary Figure 1C**). CD4+CD161+CCR7^+^CD45RA- central memory (CM) T cells accounted for ~15 and ~10%, of the CD4+CD161+ T cells, respectively, in ATB patients and healthy controls (**Figure 7B**, **Suppplementary Figure 1C**). Interestingly, CD4+CD161+T cells in ATB patients showed significantly higher expression of check-point inhibitory receptors PD-1 and Tim-3 than those in healthy controls (**Figure 7C**, **Suppplementary Figure 1C**).

Most importantly, CD4+CD161+T cells from ATB patients displayed defects in the ability to inhibit intracellular mycobacterial growth in infected hMDM (**Figure 7D**). Consistently, CD4+CD161+T cells in ATB patients showed the reduced ability to produce IFN-γ and perforin upon exposure to mycobacterium-infected hMDM (**Figure 7E**, **Suppplementary Figure 1C**), while these two cytokines proved to be critical for the CD4+CD161+T-cell mediated growth inhibition of intracellular mycobacteria (**Figure 3**). Of note, the blockade of PD1 or Tim3 resulted in ~30% decrease of BCG counts (**Suppplementary Figure 1C**).

Taken together, these results suggest thatCD4+CD161+T cells sorted from ATB patients displayed defective production of IFN-γ and perforin, leading to impaired antimicrobial activity against mycobacteria.

## Discussion

Our previous work demonstrated a role for an fast-acting CD4+ T cells in containing acute Mtb replication and extrapulmonary dissemination at the early stage of pulmonary TB infection (19), yet the identity of the protective T cell subpopulation or its mechanism of action were not elucidated. Here, we identified a fast-acting CD4+CD161+T-cell subset in unexposed healthy humans capable of rapidly producing anti-TB cytokines and inhibiting intracellular Mtb growth. Mechanistically, the fast-acting CD4+CD161+T-cell subset inhibit/kill intracellular mycobacteria in infected macrophages *via* the CD161 pathway, as well as release of perforin and IFN-γ involving autophagy distinguishing themselves from the slow adaptive/conventional CD4+ T cells not expressing CD161. These findings are clinically relevant as CD4+CD161+T-cells in TB patients exhibit a loss or reduced the ability to inhibit the growth of intracellular mycobacteria as well as to produce perforin and IFN-γ. These data define CD4+CD161+T cells as a human T cell subpopulation with the capacity to act as an early protective CD4+ T-cell subset as implicated in NHP.

Published studies have shown that CD4+ T effector cells in exposed or vaccinated individuals can express perforin/FasL and exert CTL-like activities against viral infections (38–41). Concurrently, Mtb-activated CD4+ T effector cells generated from tuberculin skin test-positive persons can mediate perforin/FasL-independent inhibition of intracellular Mtb (18). Extending those published studies, here we illustrate that anti-Mtb primary CD4+ T cells can exist even in unexposed healthy humans and that such Mtb-inhibiting primary CD4+ T cells are the CD4+CD161+ subset rather than other CD4+ T cells not expressing CD161. In addition, we also define immune mechanisms whereby the CD4+CD161+ T-cell subset activate and inhibit the growth of intracellular mycobacteria upon exposure to infected target cells. Notably, unexposed healthy donors in our study are adult humans without evidence of LTBI, and the MHC-unrestricted inhibition of Mtb by primary CD4+CD161+T cells would not be simply attributed to the decades-long memory from BCG vaccination at newborns. Moreover, memory phenotype of primary CD4+CD161+ T cells in unexposed healthy humans may not necessarily represent decades-long responses to BCG. This notion is supported by the recent reports demonstrating that pre-existing memory phenotype T effector cells with or without defined antigen specificity can be found in unexposed humans, NHP and mice [(42–44) and not shown].

CD4+CD161+T-cell subset appear to be unique in that they can recognize and inhibit/kill intracellular Mtb in infected target cells without the prior slow adaptive responses involving MHC-mediated antigen processing/presentation and subsequent TCR recognition and T-cell activation/differentiation. This notion is supported by our results demonstrating that the CD4+CD161+T-cell subset can inhibit intracellular mycobacteria in the infected MHC II-mismatched allogeneic macrophages and lung epithelia cells. On the other hand, the CD4+CD161+T-cell subset can act like other unconventional T-cell populations MAIT, NKT and Vγ2Vδ2 T cells (21, 22) to produce wide-spectrum cytokines including perforin, IFN-γ, TNF-α, and IL-17. Notably, high-frequency CD4+CD161+T cells are noted in unexposed individuals, but most of them are not the unconventional NKT cells as revealed by αGal-loaded CD1d tetramer (data not shown). It is also worth to mention that anti-TB cytokines produced by the CD4+CD161+T-cell subset can certainly contribute to protective immunity against very early Mtb infection as seen in NHP and human resisters (16, 19), while only perforin and IFN-γ are required for the ability of CD4+CD161+T-cells to inhibit the growth of intracellular mycobacteria.

Mechanisms whereby CD4+CD161+T cells inhibit intracellular mycobacteria appear to involve multiple immune events. The results in the CD161 blockade implicate that CD161 binding to the LLT1 ligand on infected macrophages can bring proximity between CD4+CD161+T cells and target cells for immune activation and subsequent anti-TB cytokine actions. In fact, previous data has shown that the interaction of LLT1 and CD161 resulted in the production of IFN-γ in T cells (36).However, this hypothesis needs more data to prove. Moreover, the mechanism of how CD4+CD161+T cells sensed mycobacteria-infected macrophages is unclear. Concurrently, the perforin-induced action may act in concert with IFN-γ-driven macrophage activation/autophagy leading to inhibiting mycobacteria (34). These hypothetical mechanisms are supported by our results derived from experiments using anti-CD161, anti-perforin or anti-IFN-γ blocking antibodies and autophagy inhibitors, respectively. It is also noteworthy that the IFN-γ action on infected macrophages can trigger IFN-γ-dependent selective autophagy leading to the control of intracellular mycobacteria (23, 34).

Given the fast-acting capabilities to produce anti-TB cytokines and inhibit intracellular mycobacteria, CD4+CD161+ T cells may play a role in protective immunity against initial Mtb infection. In fact, our recent NHP work has shown that an unconventional CD4+ T-cell subset is required to control very early Mtb dissemination after pulmonary infection (19). In addition, adoptive transfer of humanCD4+CD161+T cells can confer protective immunity against BCG infection in SCID mice. Furthermore, our data in human TB patients implicate that a loss of the ability of CD4+CD161+T cells to inhibit intracellular mycobacteria may contribute to the inefficient control of Mtb infection and development of TB.

Thus, the current study identifies and defines Mtb-inhibiting function and mechanisms of a fast-acting primary CD4+CD161+T-cell subset in unexposed healthy humans as well as dysfunction of this CD4+CD161+T-cell immunity mechanism in TB patients. Findings suggest that CD4+CD161+T-cell subset may contribute to protection or sterilizing immunity against initial Mtb infection upon exposure in humans.

## Data Availability Statement

The raw data supporting the conclusions of this article will be made available by the authors, without undue reservation.

## Ethics Statement

The studies involving human participants were reviewed and approved by Human subjects’ research and institutional biosafety committees at Shanghai Pulmonary Hospital. The patients/participants provided their written informed consent to participate in this study. The animal study was reviewed and approved by Human subjects’ research and institutional biosafety committees at Shanghai Pulmonary Hospital.

## Author Contributions

RY, HBS, WS and ZWC designed the project. RY, JP, YDL, LS, EZY, XNS, LY, YDL, and YP performed the experiments. RY, RM, HBS and ZWC analyzed the data and jointly wrote the manuscript. All authors contributed to the article and approved the submitted version.

## Funding

This work was supported by Chinese National Major Projects Grants (2018ZX10731301-006-001), National Natural Science Foundation of China (Grant No. 81901607, 31970876), Program for Outstanding Medical Academic Leader (No2019LJ13), and Clinical Research Plan of SHDC (NO.16CR1028B).

## Conflict of Interest

RY was employed by Wuhan YZY Biopharma Co., Ltd.

The remaining authors declare that the research was conducted in the absence of any commercial or financial relationships that could be construed as a potential conflict of interest.

## Supplementary Material

Supplementary Figure 1 Representative flow cytometry histograms show purity and effector functions ofCD4+CD161+T cells and controls isolated from unexposed healthy donors, as well as compare phenotypes of CD4+CD161+ T cells between ATB and HC groups and the specific staining of LC3B in BCG-infected hMDM. **(A)** Purity of cell subsets enriched by MACS methods used for mycobacteria inhibition assay. From Left to Right, enriched B cells, CD4+CD161- T cells andCD4+CD161+Tcells. **(B)**. Representative flow cytometry histograms showing production of anti-TB cytokines (IFN-γ and TNF-α) by CD4+CD161+ andCD4+CD161- T-cell subsets after PMA+Ionomycin stimulation. **(C)**. Representative flow cytometry histograms comparing frequencies ofCD4+CD161+ T cells in blood between ATB and HC groups (Top-left), and the expression patterns of CD45RA/CCR7 (Lower-left) and PD-1, Tim-3 (Right) on gatedCD4+CD161+ T cells. **(D)**. Representative flow cytometry histograms measuring perforin-producingCD4+CD161+ T cells in PBMC from ATB and HC groups stimulated with BCG-infected hMDM. **(E)**. Specific staining of LC3B puncta by LC3B Ab in hMDM. Fluorescence imaging of BCG-infected hMDM stained with rabbit-Isotype lgG Ab or rabbit LC3B Ab that was diluted at 1/100 in blocking solution for 2 h, washed three times with PBS, and incubated with Alexa fluor 488-conjugated anti-rabbit IgG Ab (1/200 in blocking solution) for 1 h. After washed with PBS, the cells were further incubated with 10 μg/ml Hocheststain for 20 min, and then used for confocal microscopy (Zeiss, German) analysis. White arrows indicate the LC3B puncta.

Supplementary Figure 2 Blockade of PD1 or Tim3 enhanced anti-mycobacteria ability of CD4+CD161+ T cells from active tuberculosis patients. CD4+CD161+ T cells from patients with active tuberculosis (n=3) were cultured with BCG-infected THP-1 cells for 3 days under the treatment of Media, Isotype, anti-PD1 or anti-Tim3 (antibodies were used at 5 ug/mL). Shown are mean survival indexes ± SD for BCG bacilli in each group. ****p < 0.0001, ***p < 0.001 vs Isotype group (ANOVA, Dunnett’s test).

## References

[1] Collaborators, G.B.D.C.o.D. Global, regional, and national age-sex specific mortality for 264 causes of death, 1980-2016: a systematic analysis for the Global Burden of Disease Study 2016. Lancet (2017) 390(10100):1151–210. 10.1016/S0140-6736(17)32152-9 PMC5605883 28919116

[2] WHO. Global Tuberculosis Report 2017, The World Health Organization Annual Repor. WHO (2017). Available at: http://www.who.int/tb/publications/global_report/gtbr2017_main_text.pdf.

[3] Colditz GA Brewer TF Berkey CS Wilson ME Burdick E Fineberg HV. Efficacy of bcg vaccine in the prevention of tuberculosis: Meta-analysis of the published literature. JAMA (1994) 271(9):698–702. 10.1001/jama.271.9.698 8309034

[4] Yang E Yang R Guo M Huang D Wang W Zhang Z. Multidrug-resistant tuberculosis (MDR-TB) strain infection in macaques results in high bacilli burdens in airways, driving broad innate/adaptive immune responses. Emerg Microbes Infect (2018) 7(1):207. 10.1038/s41426-018-0213-z 30538219 PMC6290002

[5] Modlin RL Bloom BR. TB or not TB: that is no longer the question. Sci Transl Med (2013) 5(213):213sr6. 10.1126/scitranslmed.3007402 24285487

[6] Cooper AM. Cell-mediated immune responses in tuberculosis. Annu Rev Immunol (2009) 27:393–422. 10.1146/annurev.immunol.021908.132703 19302046 PMC4298253

[7] Sakai S Mayer-Barber KD Barber DL. Defining features of protective CD4 T cell responses to Mycobacterium tuberculosis. Curr Opin Immunol (2014) 29:137–42. 10.1016/j.coi.2014.06.003 PMC4122329 25000593

[8] Gao L Zhou F Li X Jin Q. HIV/TB co-infection in mainland China: a meta-analysis. PloS One (2010) 5(5):e10736. 10.1371/journal.pone.0010736 20505769 PMC2873981

[9] Orme IM. Characteristics and specificity of acquired immunologic memory to Mycobacterium tuberculosis infection. J Immunol (1988) 140(10):3589–93. 3129497

[10] Caruso AM Serbina N Klein E Triebold K Bloom BR Flynn JL. Mice deficient in CD4 T cells have only transiently diminished levels of IFN-gamma, yet succumb to tuberculosis. J Immunol (1999) 162(9):5407–16. 10228018

[11] Scanga CA. Depletion of CD4(+) T cells causes reactivation of murine persistent tuberculosis despite continued expression of interferon gamma and nitric oxide synthase 2. J Exp Med (2000) 192(3):347–58. 10.1084/jem.192.3.347 PMC2193220 10934223

[12] O’Garra A. The immune response in tuberculosis. Annu Rev Immunol (2013) 31:475–527. 10.1146/annurev-immunol-032712-095939 23516984

[13] Khader SA. IL-23 and IL-17 in the establishment of protective pulmonary CD4+ T cell responses after vaccination and during Mycobacterium tuberculosis challenge. Nat Immunol (2007) 8(4):369–77. 10.1038/ni1449 17351619

[14] Dijkman K Sombroek CC Vervenne RAW Hofman SO Boot C Remarque EJ. Prevention of tuberculosis infection and disease by local BCG in repeatedly exposed rhesus macaques. Nat Med (2019) 25(2):255–62. 10.1038/s41591-018-0319-9 30664782

[15] Cowley SC Elkins KL. CD4+ T cells mediate IFN-gamma-independent control of Mycobacterium tuberculosis infection both in vitro and in vivo. J Immunol (2003) 171(9):4689–99. 10.4049/jimmunol.171.9.4689 14568944

[16] Sallin MA Kauffman KD Riou C Du Bruyn E Foreman TW Sakai S. Host resistance to pulmonary Mycobacterium tuberculosis infection requires CD153 expression. Nat Microbiol (2018) 3(11):1198–205. 10.1038/s41564-018-0231-6 30202016

[17] Ottenhoff TH Ab BK Van Embden JD Thole JE Kiessling R. The recombinant 65-kD heat shock protein of Mycobacterium bovis Bacillus Calmette-Guerin/M. tuberculosis is a target molecule for CD4+ cytotoxic T lymphocytes that lyse human monocytes. J Exp Med (1988) 168(5):1947–52. 10.1084/jem.168.5.1947 PMC2189100 2903217

[18] Canaday DH Wilkinson RD Li Q Harding CV Silver RF Boom WH. CD4(+) and CD8(+) T cells kill intracellular Mycobacterium tuberculosis by a perforin and Fas/Fas ligand-independent mechanism. J Immunol (2001) 167(5):2734–42. 10.4049/jimmunol.167.5.2734 11509617

[19] Yao S Huang D Chen CY Halliday L Wang RC Chen ZW. CD4+ T cells contain early extrapulmonary tuberculosis (TB) dissemination and rapid TB progression and sustain multieffector functions of CD8+ T and CD3- lymphocytes: mechanisms of CD4+ T cell immunity. J Immunol (2014) 192(5):2120–32. 10.4049/jimmunol.1301373 PMC4104690 24489088

[20] Shen L Huang D Qaqish A Frencher J Yang R Shen H. Fast-acting γδ T-cell subpopulation and protective immunity against infections. Immunol Rev (2020) 298(1):254–63. 10.1111/imr.12927 33037700

[21] Fergusson JR Smith KE Fleming VM Rajoriya N Newell EW Simmons R. CD161 defines a transcriptional and functional phenotype across distinct human T cell lineages. Cell Rep (2014) 9(3):1075–88. 10.1016/j.celrep.2014.09.045 PMC4250839 25437561

[22] Gao Y Williams AP. Role of Innate T Cells in Anti-Bacterial Immunity. Front Immunol (2015) 6:302. 10.3389/fimmu.2015.00302 26124758 PMC4463001

[23] Yang R Yang E Shen L Modlin RL Shen H Chen ZW. IL-12+IL-18 Cosignaling in Human Macrophages and Lung Epithelial Cells Activates Cathelicidin and Autophagy, Inhibiting Intracellular Mycobacterial Growth. J Immunol (2018) 200(7):2405–17. 10.4049/jimmunol.1701073 PMC5860987 29453279

[24] Shen H Gu J Xiao H Liang S Yang E Yang R. Selective Destruction of Interleukin 23-Induced Expansion of a Major Antigen-Specific gammadelta T-Cell Subset in Patients With Tuberculosis. J Infect Dis (2017) 215(3):420–30. 10.1093/infdis/jiw511 PMC5853380 27789724

[25] Yang R Yao L Shen L Sha W Modlin RL Shen H. IL-12 Expands and Differentiates Human Vgamma2Vdelta2 T Effector Cells Producing Antimicrobial Cytokines and Inhibiting Intracellular Mycobacterial Growth. Front Immunol (2019) 10:913. 10.3389/fimmu.2019.01742 31080452 PMC6497761

[26] Afzali B Mitchell PJ Edozie FC Povoleri GA Dowson SE Demandt L. CD161 expression characterizes a subpopulation of human regulatory T cells that produces IL-17 in a STAT3-dependent manner. Eur J Immunol (2013) 43(8):2043–54. 10.1002/eji.201243296 PMC3815561 23677517

[27] Pesenacker AM Bending D Ursu S Wu Q Nistala K Wedderburn LR. CD161 defines the subset of FoxP3+ T cells capable of producing proinflammatory cytokines. Blood (2013) 121(14):2647–58. 10.1182/blood-2012-08-443473 PMC3617631 23355538

[28] Qiu Y Chen J Liao H Zhang Y Wang H Li S. Tim-3-expressing CD4+ and CD8+ T cells in human tuberculosis (TB) exhibit polarized effector memory phenotypes and stronger anti-TB effector functions. PloS Pathog (2012) 8(11):e1002984. 10.1371/journal.ppat.1002984 23144609 PMC3493466

[29] Chen CY Yao S Huang D Wei H Sicard H Zeng G. Phosphoantigen/IL2 expansion and differentiation of Vgamma2Vdelta2 T cells increase resistance to tuberculosis in nonhuman primates. PloS Pathog (2013) 9(8):e1003501. 10.1371/journal.ppat.1003501 23966854 PMC3744401

[30] Spencer CT Abate G Sakala IG Xia M Truscott SM Eickhoff CS. Granzyme A produced by gamma(9)delta(2) T cells induces human macrophages to inhibit growth of an intracellular pathogen. PloS Pathog (2013) 9(1):e1003119. 10.1371/journal.ppat.1003119 23326234 PMC3542113

[31] Qaqish A Huang D Chen CY Zhang Z Wang R Li S. Adoptive Transfer of Phosphoantigen-Specific gammadelta T Cell Subset Attenuates Mycobacterium tuberculosis Infection in Nonhuman Primates. J Immunol (2017) 198(12):4753–63. 10.4049/jimmunol.1602019 PMC5557270 28526681

[32] Maggi L Santarlasci V Capone M Peired A Frosali F Crome SQ. CD161 is a marker of all human IL-17-producing T-cell subsets and is induced by RORC. Eur J Immunol (2010) 40(8):2174–81. 10.1002/eji.200940257 20486123

[33] Cosmi L De Palma R Santarlasci V Maggi L Capone M Frosali F. Human interleukin 17-producing cells originate from a CD161+CD4+ T cell precursor. J Exp Med (2008) 205(8):1903–16. 10.1084/jem.20080397 PMC2525581 18663128

[34] Fabri M Stenger S Shin DM Yuk JM Liu PT Realegeno S. Vitamin D is required for IFN-gamma-mediated antimicrobial activity of human macrophages. Sci Transl Med (2011) 3(104):104ra102. 10.1126/scitranslmed.3003045 PMC3269210 21998409

[35] Seglen PO Gordon PB. 3-Methyladenine: specific inhibitor of autophagic/lysosomal protein degradation in isolated rat hepatocytes. Proc Natl Acad Sci U.S.A. (1982) 79(6):1889–92. 10.1073/pnas.79.6.1889 PMC346086 6952238

[36] Aldemir H Prod’homme V Dumaurier MJ Retiere C Poupon G Cazareth J. Cutting edge: lectin-like transcript 1 is a ligand for the CD161 receptor. J Immunol (2005) 175(12):7791–5. 10.4049/jimmunol.175.12.7791 16339512

[37] Yang Q Xu Q Chen Q Li J Zhang M Cai Y. Discriminating Active Tuberculosis from Latent Tuberculosis Infection by flow cytometric measurement of CD161-expressing T cells. Sci Rep (2015) 5:17918. 10.1038/srep17918 26643453 PMC4672319

[38] Zaunders JJ Dyer WB Wang B Munier ML Miranda-Saksena M Newton R. Identification of circulating antigen-specific CD4+ T lymphocytes with a CCR5+, cytotoxic phenotype in an HIV-1 long-term nonprogressor and in CMV infection. Blood (2004) 103(6):2238–47. 10.1182/blood-2003-08-2765 14645006

[39] Brown DM Kamperschroer C Dilzer AM Roberts DM Swain SL. IL-2 and antigen dose differentially regulate perforin- and FasL-mediated cytolytic activity in antigen specific CD4+ T cells. Cell Immunol (2009) 257(1-2):69–79. 10.1016/j.cellimm.2009.03.002 19338979 PMC2683476

[40] Brown DM Lee S Garcia-Hernandez Mde L Swain SL. Multifunctional CD4 cells expressing gamma interferon and perforin mediate protection against lethal influenza virus infection. J Virol (2012) 86(12):6792–803. 10.1128/JVI.07172-11 PMC3393557 22491469

[41] Takeuchi A Saito T. CD4 CTL, a Cytotoxic Subset of CD4(+) T Cells, Their Differentiation and Function. Front Immunol (2017) 8:194. 10.3389/fimmu.2017.00194 28280496 PMC5321676

[42] Jameson SC Masopust D. Diversity in T cell memory: an embarrassment of riches. Immunity (2009) 31(6):859–71. 10.1016/j.immuni.2009.11.007 PMC2957815 20064446

[43] Pepper M Jenkins MK. Origins of CD4(+) effector and central memory T cells. Nat Immunol (2011) 12(6):467–71. 10.1038/ni.2038 PMC4212218 21739668

[44] Su LF Kidd BA Han A Kotzin JJ Davis MM. Virus-specific CD4(+) memory-phenotype T cells are abundant in unexposed adults. Immunity (2013) 38(2):373–83. 10.1016/j.immuni.2012.10.021 PMC3626102 23395677

