# Supplementary figures and images for: A CD4+CD161+ T-Cell Subset Present in Unexposed Humans, Not Tb Patients, Are Fast Acting Cells That Inhibit the Growth of Intracellular Mycobacteria Involving CD161 Pathway, Perforin, and IFN-γ/Autophagy
Source: Front Immunol. 2021 Feb 26;12:599641. doi: 10.3389/fimmu.2021.599641 (PMC7959736; doi:10.3389/fimmu.2021.599641)

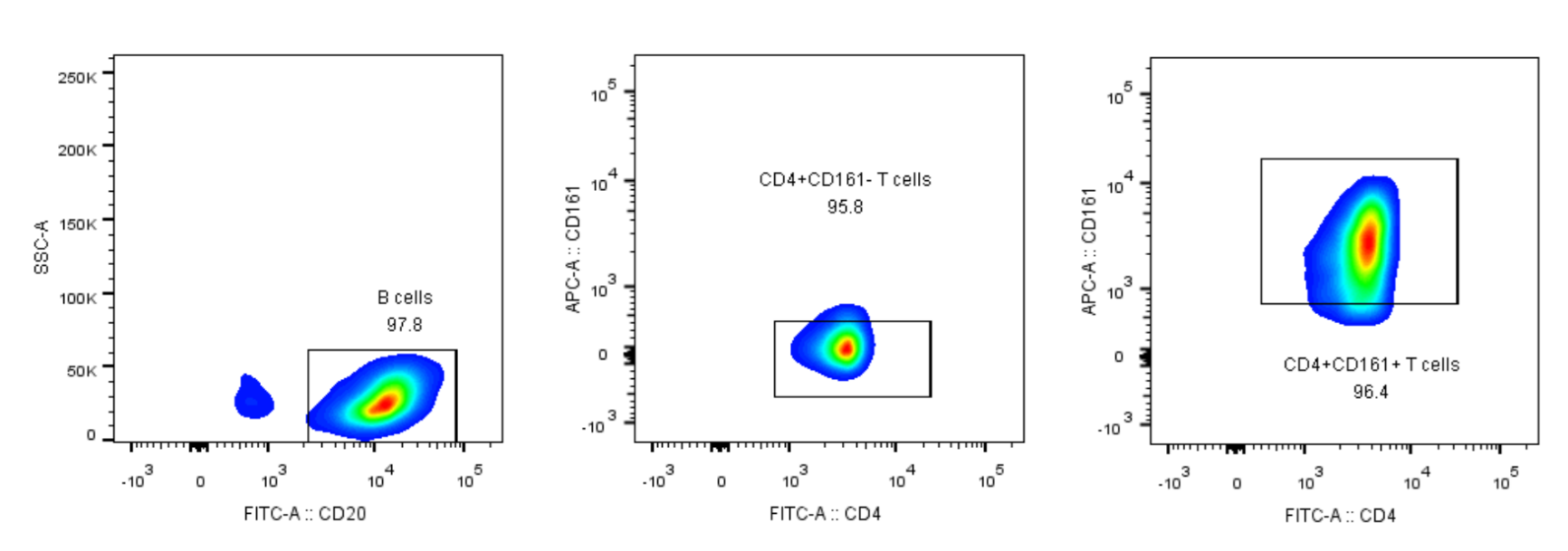

Supplement: Supplementary Figure 1 — Representative flow cytometry histograms show purity and effector functions ofCD4+CD161+T cells and controls isolated from unexposed healthy donors, as well as compare phenotypes of CD4+CD161+ T cells between ATB and HC groups and the specific staining of LC3B in BCG-infected hMDM. (A) Purity of cell subsets enriched by MACS methods used for mycobacteria inhibition assay. From Left to Right, enriched B cells, CD4+CD161- T cells andCD4+CD161+Tcells. (B). Representative flow cytometry histograms showing production of anti-TB cytokines (IFN-γ and TNF-α) by CD4+CD161+ andCD4+CD161- T-cell subsets after PMA+Ionomycin stimulation. (C). Representative flow cytometry histograms comparing frequencies ofCD4+CD161+ T cells in blood between ATB and HC groups (Top-left), and the expression patterns of CD45RA/CCR7 (Lower-left) and PD-1, Tim-3 (Right) on gatedCD4+CD161+ T cells. (D). Representative flow cytometry histograms measuring perforin-producingCD4+CD161+ T cells in PBMC from ATB and HC groups stimulated with BCG-infected hMDM. (E). Specific staining of LC3B puncta by LC3B Ab in hMDM. Fluorescence imaging of BCG-infected hMDM stained with rabbit-Isotype lgG Ab or rabbit LC3B Ab that was diluted at 1/100 in blocking solution for 2 h, washed three times with PBS, and incubated with Alexa fluor 488-conjugated anti-rabbit IgG Ab (1/200 in blocking solution) for 1 h. After washed with PBS, the cells were further incubated with 10 μg/ml Hocheststain for 20 min, and then used for confocal microscopy (Zeiss, German) analysis. White arrows indicate the LC3B puncta. [file DataSheet_1.zip › Supplementary Figure 1A.TIF]

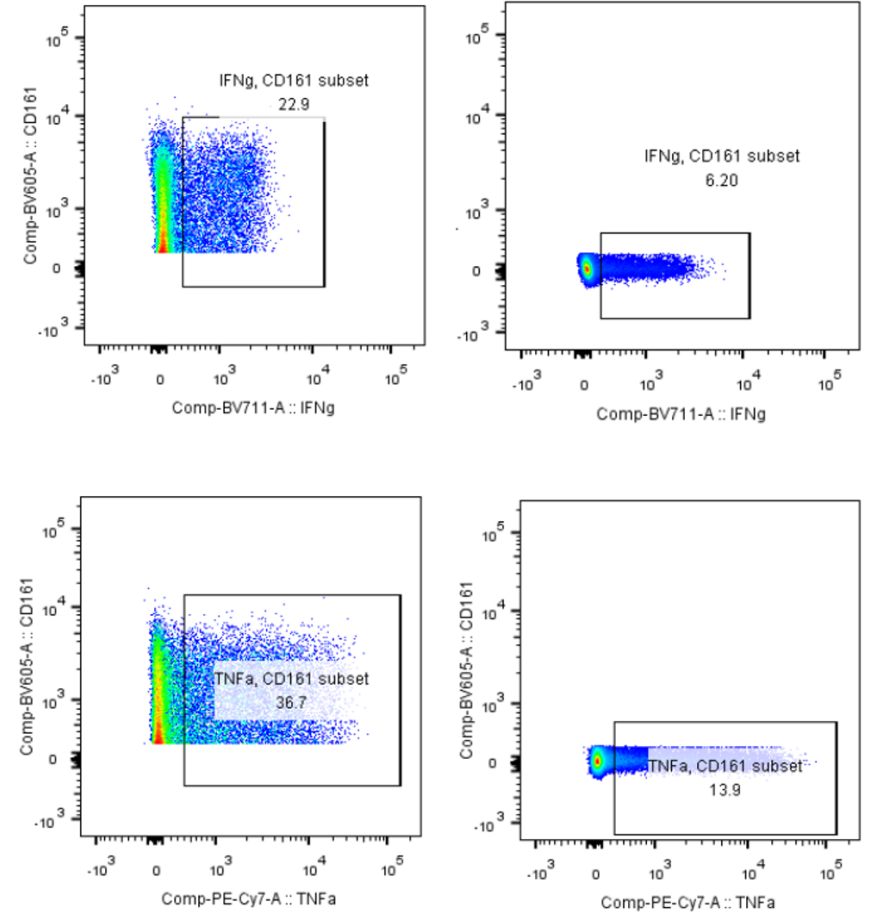

Supplement: Supplementary Figure 1 — Representative flow cytometry histograms show purity and effector functions ofCD4+CD161+T cells and controls isolated from unexposed healthy donors, as well as compare phenotypes of CD4+CD161+ T cells between ATB and HC groups and the specific staining of LC3B in BCG-infected hMDM. (A) Purity of cell subsets enriched by MACS methods used for mycobacteria inhibition assay. From Left to Right, enriched B cells, CD4+CD161- T cells andCD4+CD161+Tcells. (B). Representative flow cytometry histograms showing production of anti-TB cytokines (IFN-γ and TNF-α) by CD4+CD161+ andCD4+CD161- T-cell subsets after PMA+Ionomycin stimulation. (C). Representative flow cytometry histograms comparing frequencies ofCD4+CD161+ T cells in blood between ATB and HC groups (Top-left), and the expression patterns of CD45RA/CCR7 (Lower-left) and PD-1, Tim-3 (Right) on gatedCD4+CD161+ T cells. (D). Representative flow cytometry histograms measuring perforin-producingCD4+CD161+ T cells in PBMC from ATB and HC groups stimulated with BCG-infected hMDM. (E). Specific staining of LC3B puncta by LC3B Ab in hMDM. Fluorescence imaging of BCG-infected hMDM stained with rabbit-Isotype lgG Ab or rabbit LC3B Ab that was diluted at 1/100 in blocking solution for 2 h, washed three times with PBS, and incubated with Alexa fluor 488-conjugated anti-rabbit IgG Ab (1/200 in blocking solution) for 1 h. After washed with PBS, the cells were further incubated with 10 μg/ml Hocheststain for 20 min, and then used for confocal microscopy (Zeiss, German) analysis. White arrows indicate the LC3B puncta. [file DataSheet_1.zip › Supplementary Figure 1B.TIF]

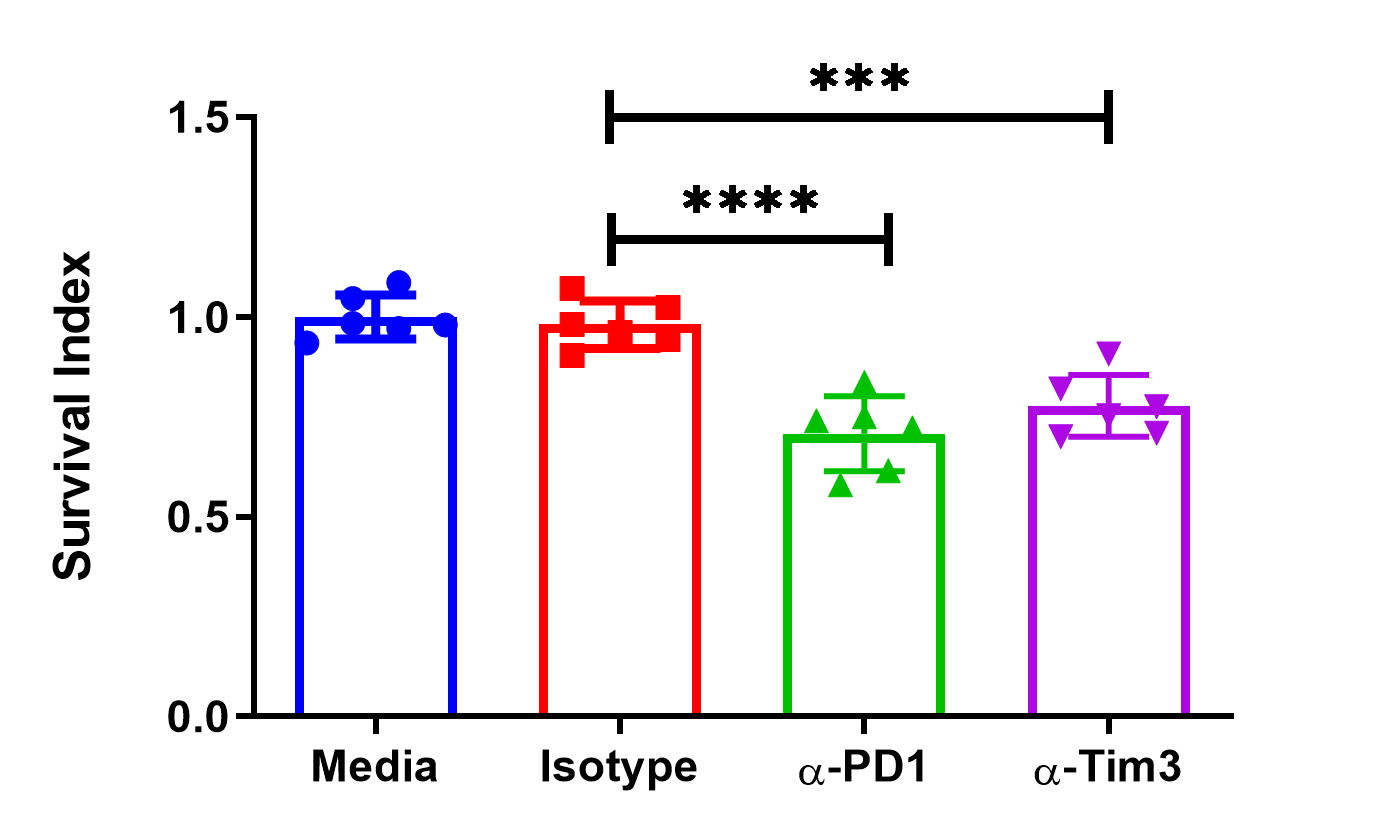

Supplement: Supplementary Figure 1 — Representative flow cytometry histograms show purity and effector functions ofCD4+CD161+T cells and controls isolated from unexposed healthy donors, as well as compare phenotypes of CD4+CD161+ T cells between ATB and HC groups and the specific staining of LC3B in BCG-infected hMDM. (A) Purity of cell subsets enriched by MACS methods used for mycobacteria inhibition assay. From Left to Right, enriched B cells, CD4+CD161- T cells andCD4+CD161+Tcells. (B). Representative flow cytometry histograms showing production of anti-TB cytokines (IFN-γ and TNF-α) by CD4+CD161+ andCD4+CD161- T-cell subsets after PMA+Ionomycin stimulation. (C). Representative flow cytometry histograms comparing frequencies ofCD4+CD161+ T cells in blood between ATB and HC groups (Top-left), and the expression patterns of CD45RA/CCR7 (Lower-left) and PD-1, Tim-3 (Right) on gatedCD4+CD161+ T cells. (D). Representative flow cytometry histograms measuring perforin-producingCD4+CD161+ T cells in PBMC from ATB and HC groups stimulated with BCG-infected hMDM. (E). Specific staining of LC3B puncta by LC3B Ab in hMDM. Fluorescence imaging of BCG-infected hMDM stained with rabbit-Isotype lgG Ab or rabbit LC3B Ab that was diluted at 1/100 in blocking solution for 2 h, washed three times with PBS, and incubated with Alexa fluor 488-conjugated anti-rabbit IgG Ab (1/200 in blocking solution) for 1 h. After washed with PBS, the cells were further incubated with 10 μg/ml Hocheststain for 20 min, and then used for confocal microscopy (Zeiss, German) analysis. White arrows indicate the LC3B puncta. [file DataSheet_1.zip › Supplementary Figure 1C.TIF]

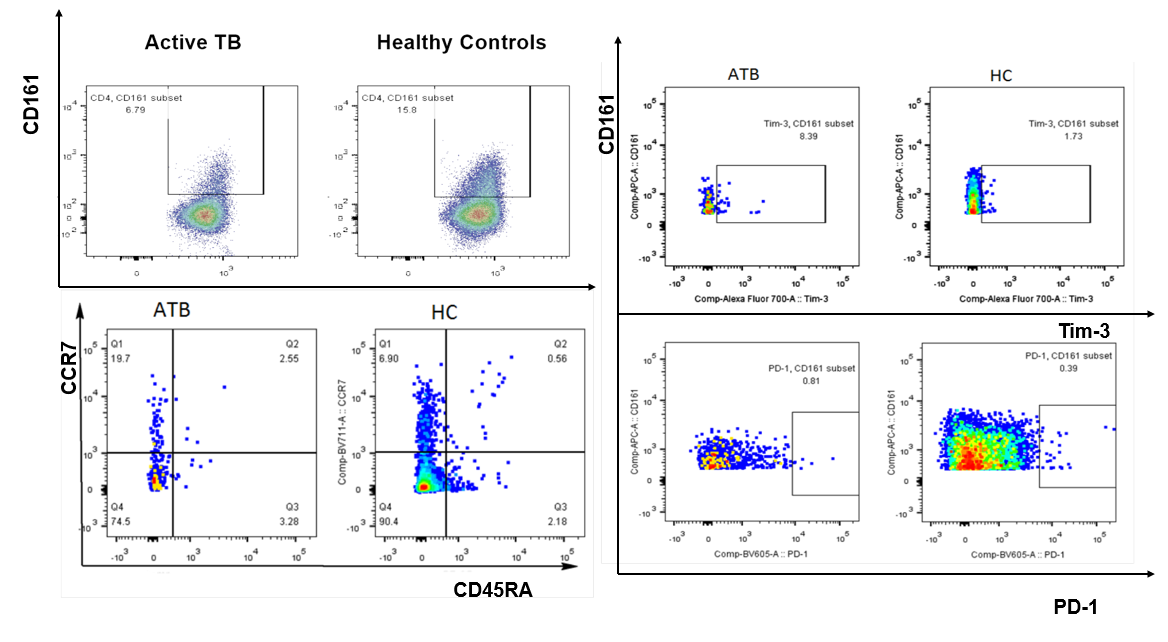

Supplement: Supplementary Figure 1 — Representative flow cytometry histograms show purity and effector functions ofCD4+CD161+T cells and controls isolated from unexposed healthy donors, as well as compare phenotypes of CD4+CD161+ T cells between ATB and HC groups and the specific staining of LC3B in BCG-infected hMDM. (A) Purity of cell subsets enriched by MACS methods used for mycobacteria inhibition assay. From Left to Right, enriched B cells, CD4+CD161- T cells andCD4+CD161+Tcells. (B). Representative flow cytometry histograms showing production of anti-TB cytokines (IFN-γ and TNF-α) by CD4+CD161+ andCD4+CD161- T-cell subsets after PMA+Ionomycin stimulation. (C). Representative flow cytometry histograms comparing frequencies ofCD4+CD161+ T cells in blood between ATB and HC groups (Top-left), and the expression patterns of CD45RA/CCR7 (Lower-left) and PD-1, Tim-3 (Right) on gatedCD4+CD161+ T cells. (D). Representative flow cytometry histograms measuring perforin-producingCD4+CD161+ T cells in PBMC from ATB and HC groups stimulated with BCG-infected hMDM. (E). Specific staining of LC3B puncta by LC3B Ab in hMDM. Fluorescence imaging of BCG-infected hMDM stained with rabbit-Isotype lgG Ab or rabbit LC3B Ab that was diluted at 1/100 in blocking solution for 2 h, washed three times with PBS, and incubated with Alexa fluor 488-conjugated anti-rabbit IgG Ab (1/200 in blocking solution) for 1 h. After washed with PBS, the cells were further incubated with 10 μg/ml Hocheststain for 20 min, and then used for confocal microscopy (Zeiss, German) analysis. White arrows indicate the LC3B puncta. [file DataSheet_1.zip › Supplementary Figure 1D.TIF]

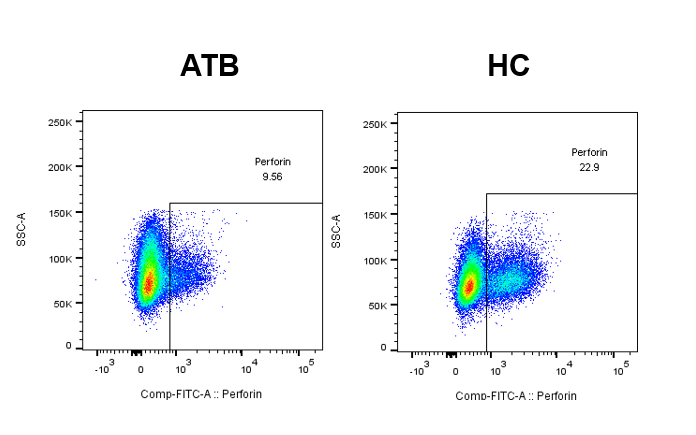

Supplement: Supplementary Figure 1 — Representative flow cytometry histograms show purity and effector functions ofCD4+CD161+T cells and controls isolated from unexposed healthy donors, as well as compare phenotypes of CD4+CD161+ T cells between ATB and HC groups and the specific staining of LC3B in BCG-infected hMDM. (A) Purity of cell subsets enriched by MACS methods used for mycobacteria inhibition assay. From Left to Right, enriched B cells, CD4+CD161- T cells andCD4+CD161+Tcells. (B). Representative flow cytometry histograms showing production of anti-TB cytokines (IFN-γ and TNF-α) by CD4+CD161+ andCD4+CD161- T-cell subsets after PMA+Ionomycin stimulation. (C). Representative flow cytometry histograms comparing frequencies ofCD4+CD161+ T cells in blood between ATB and HC groups (Top-left), and the expression patterns of CD45RA/CCR7 (Lower-left) and PD-1, Tim-3 (Right) on gatedCD4+CD161+ T cells. (D). Representative flow cytometry histograms measuring perforin-producingCD4+CD161+ T cells in PBMC from ATB and HC groups stimulated with BCG-infected hMDM. (E). Specific staining of LC3B puncta by LC3B Ab in hMDM. Fluorescence imaging of BCG-infected hMDM stained with rabbit-Isotype lgG Ab or rabbit LC3B Ab that was diluted at 1/100 in blocking solution for 2 h, washed three times with PBS, and incubated with Alexa fluor 488-conjugated anti-rabbit IgG Ab (1/200 in blocking solution) for 1 h. After washed with PBS, the cells were further incubated with 10 μg/ml Hocheststain for 20 min, and then used for confocal microscopy (Zeiss, German) analysis. White arrows indicate the LC3B puncta. [file DataSheet_1.zip › Supplementary Figure 1E.TIF]

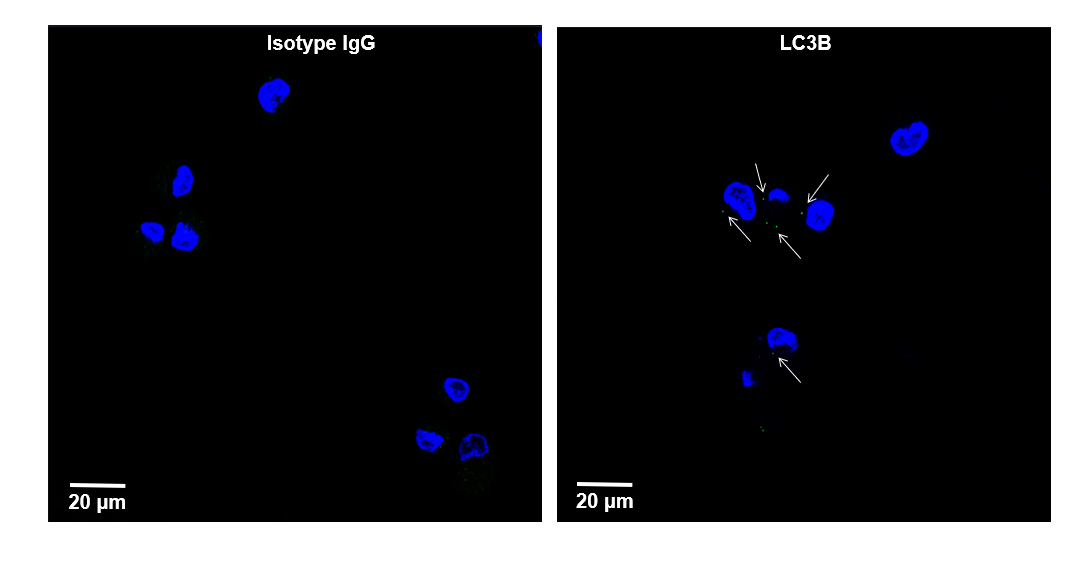

Supplement: Supplementary Figure 1 — Representative flow cytometry histograms show purity and effector functions ofCD4+CD161+T cells and controls isolated from unexposed healthy donors, as well as compare phenotypes of CD4+CD161+ T cells between ATB and HC groups and the specific staining of LC3B in BCG-infected hMDM. (A) Purity of cell subsets enriched by MACS methods used for mycobacteria inhibition assay. From Left to Right, enriched B cells, CD4+CD161- T cells andCD4+CD161+Tcells. (B). Representative flow cytometry histograms showing production of anti-TB cytokines (IFN-γ and TNF-α) by CD4+CD161+ andCD4+CD161- T-cell subsets after PMA+Ionomycin stimulation. (C). Representative flow cytometry histograms comparing frequencies ofCD4+CD161+ T cells in blood between ATB and HC groups (Top-left), and the expression patterns of CD45RA/CCR7 (Lower-left) and PD-1, Tim-3 (Right) on gatedCD4+CD161+ T cells. (D). Representative flow cytometry histograms measuring perforin-producingCD4+CD161+ T cells in PBMC from ATB and HC groups stimulated with BCG-infected hMDM. (E). Specific staining of LC3B puncta by LC3B Ab in hMDM. Fluorescence imaging of BCG-infected hMDM stained with rabbit-Isotype lgG Ab or rabbit LC3B Ab that was diluted at 1/100 in blocking solution for 2 h, washed three times with PBS, and incubated with Alexa fluor 488-conjugated anti-rabbit IgG Ab (1/200 in blocking solution) for 1 h. After washed with PBS, the cells were further incubated with 10 μg/ml Hocheststain for 20 min, and then used for confocal microscopy (Zeiss, German) analysis. White arrows indicate the LC3B puncta. [file DataSheet_1.zip › Supplementary Figure 2.TIF]
